# Supplementary material for: Evidence supported by Mendelian randomization: impact on inflammatory factors in knee osteoarthritis
Source: Front Med (Lausanne). 2024 May 28;11:1382836. doi: 10.3389/fmed.2024.1382836 (PMC11165061; doi:10.3389/fmed.2024.1382836)
Supplement: Supplementary file 1 [file Data_Sheet_1.ZIP › data/GCST90274813_5e6/813.leaveoneout.pdf]

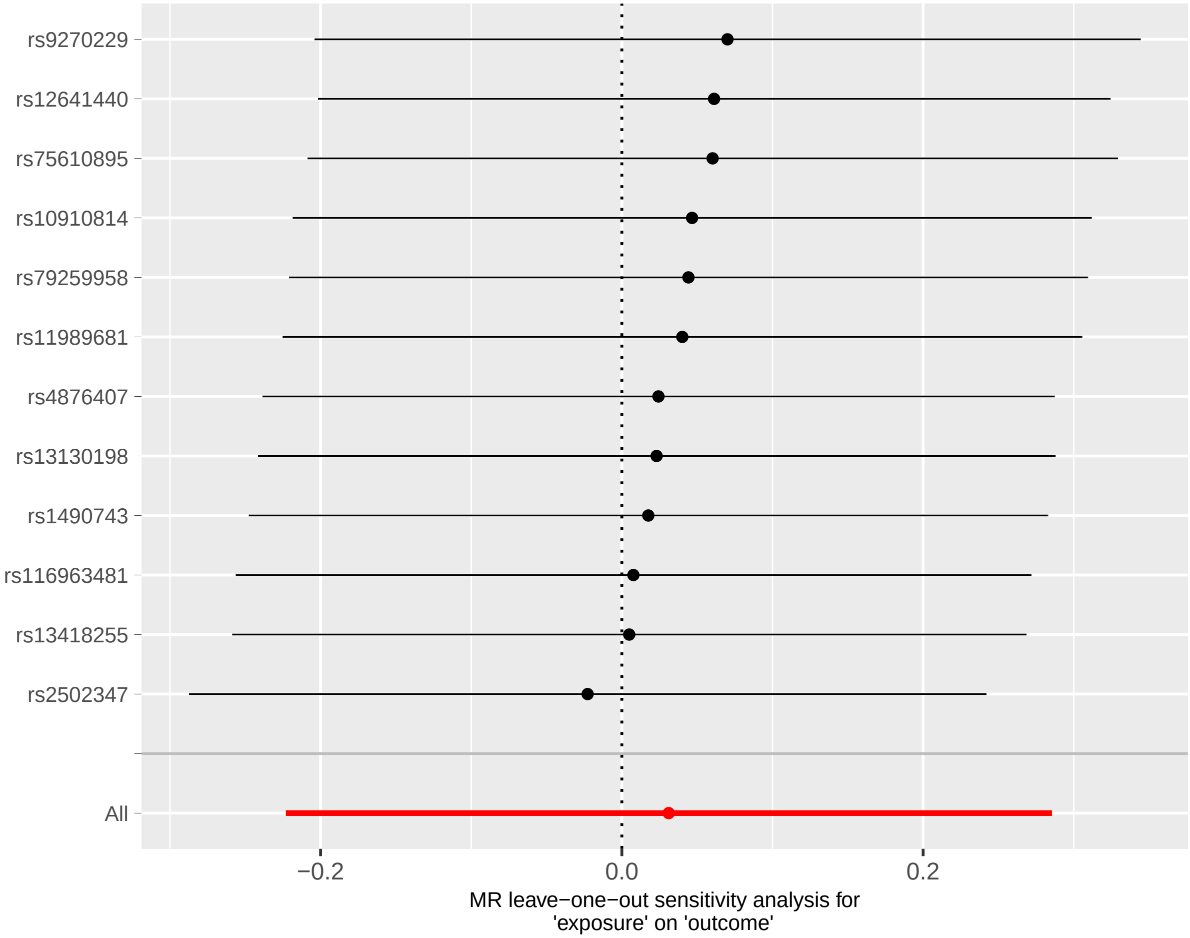
rs9270229
rs12641440
rs75610895
rs10910814
rs79259958
rs11989681
rs4876407
rs13130198
rs1490743
rs116963481
rs13418255
rs2502347
All
−0.2
0.0
0.2
MR leave−one−out sensitivity analysis for
'exposure' on 'outcome'
